# Detection of Selection Signatures Among Brazilian, Sri Lankan, and Egyptian Chicken Populations Under Different Environmental Conditions

**DOI:** 10.3389/fgene.2018.00737

**Published:** 2019-01-14

**Authors:** Muhammed Walugembe, Francesca Bertolini, Chandraratne Mahinda B. Dematawewa, Matheus P. Reis, Ahmed R. Elbeltagy, Carl J. Schmidt, Susan J. Lamont, Max F. Rothschild

**Affiliations:** ^1^Department of Animal Science, Iowa State University, Ames, IA, United States; ^2^Department of Animal Science, Faculty of Agriculture, University of Peradeniya, Kandy, Sri Lanka; ^3^Department of Animal Science, College of Agricultural and Veterinary Sciences, São Paulo State University, Jaboticabal, Brazil; ^4^Department of Animal Biotechnology, Animal Production Research Institute, Giza, Egypt; ^5^Animal and Food Sciences, University of Delaware, Newark, DE, United States

**Keywords:** chickens, environment, selection signatures, adaptation, immune system

## Abstract

Extreme environmental conditions are a major challenge in livestock production. Changes in climate, particularly those that contribute to weather extremes like drought or excessive humidity, may result in reduced performance and reproduction and could compromise the animal’s immune function. Animal survival within extreme environmental conditions could be in response to natural selection and to artificial selection for production traits that over time together may leave selection signatures in the genome. The aim of this study was to identify selection signatures that may be involved in the adaptation of indigenous chickens from two different climatic regions (Sri Lanka = Tropical; Egypt = Arid) and in non-indigenous chickens that derived from human migration events to the generally tropical State of São Paulo, Brazil. To do so, analyses were conducted using fixation index (Fst) and hapFLK analyses. Chickens from Brazil (*n* = 156), Sri Lanka (*n* = 92), and Egypt (*n* = 96) were genotyped using the Affymetrix Axiom^®^600k Chicken Genotyping Array. Pairwise Fst analyses among countries did not detect major regions of divergence between chickens from Sri Lanka and Brazil, with ecotypes/breeds from Brazil appearing to be genetically related to Asian-Indian (Sri Lanka) ecotypes. However, several differences were detected in comparisons of Egyptian with either Sri Lankan or Brazilian populations, and common regions of difference on chromosomes 2, 3 and 8 were detected. The hapFLK analyses for the three separate countries suggested unique regions that are potentially under selection on chromosome 1 for all three countries, on chromosome 4 for Sri Lankan, and on chromosomes 3, 5, and 11 for the Egyptian populations. Some of identified regions under selection with hapFLK analyses contained genes such as *TLR3, SOCS2, EOMES*, and *NFAT5* whose biological functions could provide insights in understanding adaptation mechanisms in response to arid and tropical environments.

## Introduction

Extreme environmental conditions are a major challenge in livestock production. Changes in climate, particularly those that contribute to weather extremes like drought or extreme temperatures or humidity may result in reduced performance, reproduction and could compromise the animal’s immune function (St-Pierre et al., 2003). In chickens, extreme environmental temperatures lead to generation of reactive oxygen species (ROS), causing oxidative stress and lipid peroxidation (Altan et al., 2003). However, chickens particularly the local (indigenous) breeds often adapt over time to tolerate extreme challenging environments. Local chicken populations are characterized in terms of production status by limited management and veterinary services but are considered important genetic resources. They are reported to have been derived after many hundreds of years of successful adaptations to extreme environments (Hall and Bradley, 1995). In Egypt, there is undisputed evidence that chickens (domestic fowls) were kept since 1840 B.C (Coltherd, 1966), and Egypt was a major entry of Indian chickens to the African continent (Eltanany and Hemeda, 2016; Osman et al., 2016). Egyptian local breeds are generally characterized into three groups: the first group are the native breeds such as Fayoumi and Dandarawi, second group includes the Baladi and Sinai strains, and third group results from the cross between exotic and local strains accompanied by various trait selection (Osman et al., 2016). The native/local breeds/ecotypes have been kept as backyard or free-range chickens and could have developed adaptation mechanisms to their respective climates. In spite of successful adaptations to their environments, there is limited knowledge about genomic regions involved in the adaptation of local village chickens to the specific environmental conditions. There is also uncertainty whether geographical locations of local chicken populations could be the cause of their genetic differentiation (Mahammi et al., 2016). Domestication by humans and subsequent breed formation has led to chickens being adapted in physiology, morphology, fertility, and behavior to increase production (Ericsson et al., 2014). Selection pressure, natural or artificial, has been influential in enabling chickens to adapt to their environments and may leave signatures of selection in chicken population genomes. Signatures of selection, or selective sweeps as they are sometimes called, are particular patterns of DNA that are identified in regions of the genome with mutation or have been under selection pressure in a population (Qanbari and Simianer, 2014). Larger homozygosity regions are exhibited in such regions than expected under Hardy-Weinberg equilibrium whenever there is positive selection for a particular allele. These regions may have genes with functional importance in particular processes and reflect allelic selection under differing environmental conditions.

There are many methods used in the detection of selection signatures in the genome. These methods are classified into intra-population and inter-populations statistics. Inter-population statistical analyses can be categorized into single site or haplotype differentiation analyses (Qanbari and Simianer, 2014). To detect regions of divergence or similarity, most studies have used the single site differentiation statistic commonly known as Fixation Index, Fst (Elferink et al., 2012; Gholami et al., 2015; Fleming et al., 2017) and hapFLK (Gholami et al., 2015) analyses to detect selection signatures in both commercial and non-commercial breeds. Inter-population statistics are reported to have more statistical power to detect selection signatures in recently diverged populations (Yi et al., 2010). The major concern with Fst is that it assumes the populations have same effective population size and are derived independently from one ancestral population (Price et al., 2010). HapFLK is a method that is based on extension of the FLK statistic and accounts for both the hierarchical structure and haplotype information, and its use greatly improves the detection power and can detect signatures of selection that may be occurring across several populations (Fariello et al., 2013).

In this study we applied both Fst and hapFLK statistical analyses on indigenous chicken breed/ecotype populations from three countries that have different climates [Brazil and Sri Lanka = Tropical, and Egypt = Arid] for regions where selection may have taken place and shaped the genome to enable the chickens to adapt to different environments.

## Materials and Methods

Chicken blood sample collections procedures in Brazil were approved by Animal Care and Use Committee of São Paulo State University (Process 009999/14; approved on 06 June 2014). Chicken blood samples from Egypt and Sri Lanka were collected in accordance with the local veterinary guidelines.

### Sample Collection

Blood samples were collected from 156 Brazilian, 92 Sri Lankan, and 96 Egyptian chickens under veterinary supervision in the home countries and according to accepted animal care practices. The Brazilian chickens represented eleven ecotypes/breeds (Sedosa, Cochinchina, Ketros Oceania, Suri, Backyard Giant Indian, Shamo, Brahman, Backyard, Bantham, Brazilian Musician, and Bakiva) and were sampled from different farms, outside Porto Ferreira in the State of São Paulo. A total of 92 samples were collected from 3 Sri Lanka ecotypes which were made up of 27, 34, and 31 samples collected from Gannoruwa (GN) town, Karuwalagaswewa (KR), and Uda Peradeniya (UPA) villages, respectively. A total of 95 samples were collected from an Egyptian ecotype and two breeds; 31 Baladi (Bal, ecotype) from 3 villages in Qalyubia, 31 Fayoumi (Fay) from 4 villages in Mid-Egypt, and 33 Dandarawi (Dan), from 4 villages in Southern Egypt.

### Genotyping and Quality Control

Genotyping for all samples was conducted at GeneSeek (Lincoln, NE, United States) using the Affymetrix Axiom^®^600k Array. SNP (single nucleotide polymorphism) genotype data quality filtering was assessed with PLINK 1.9 software (Chang et al., 2015) and only autosomal SNPs were screened based on parameters of >90% call rate (-geno 0.1) and minor allele frequency (MAF) > 0.02. In total, 523,186 SNPs were utilized for downstream analysis.

### Population Stratification Analyses

Multi-dimensional scaling (MDS) was performed to examine population structure for stratification in two dimensions using cluster algorithm in PLINK v1.9 (Chang et al., 2015). Shared ancestry, with no prior knowledge on the origin of the breeds, was explored using the Admixture software (Alexander et al., 2009) for varying *K*-values, ranging from 1 to 12, where *K* is the number of expected subpopulations. The optimum *K*-value of *K* = 10 was determined based on the lowest value of the cross-validation error.

### Fst Analyses

The Fst statistic analysis is a widely used approach and was performed to determine genetic differentiations between populations (Barreiro et al., 2008; Bonhomme et al., 2010; Fariello et al., 2013). Three pairwise comparisons were performed in Plink v1.9 (Purcell et al., 2007) for Brazil vs. Egypt, Sri Lanka vs. Egypt, and Brazil vs. Sri Lanka ecotypes to identify any genomic regions under increasing differentiation using an overlapping sliding window approach. The populations were designated as a case or control category based on hypothesized proxy climatic phenotype of tropical (Brazil and Sri Lanka) vs. arid (Egypt) climatic conditions. For each comparison, mean Fst (mFst) value was calculated in 100 kb sliding windows with a step size of 50 kb to examine data with 50% overlap using an in-house script (Karlsson et al., 2007). Genomic regions with the highest peaks, 0.2% of the empirical distributions of the mFst values, were considered for downstream analyses.

### HapFLK Analyses

The hapFLK statistic accounts for varying effective population sizes and haplotype structure of the populations using multi-point linkage disequilibrium model (Scheet and Stephens, 2006; Bonhomme et al., 2010; Fariello et al., 2013). This approach was used to identify possible regions under selection across chicken breeds/ecotypes within each country. To do this, it required estimation of a neighbor joining tree and a kinship matrix based on the matrix of Reynolds’ genetic distances between ecotypes/breeds (Bonhomme et al., 2010). A phylogenetic tree was constructed among the populations from the three countries: Sri Lanka (KR, UPA, and GN), Brazil (Sedosa, Cochinchina, Ketros Oceania, Suri, Backyard Giant Indian, Shamo, Brahman, Backyard, Bantham, Brazilian Musician, Bakiva), and Egypt [Baladi (Bal), Fayoumi (Fay), and Dandarawi (Dan)]. To identify any regions under selection, analyses were performed separately across breeds/ecotypes within each climatic region (country). The number of haplotype clusters per chromosome was determined in fastPHASE using cross-validation based estimation and was set at 15 (Scheet and Stephens, 2006). The hapFLK values were generated for each SNP and computation of P-values were performed using a chi-square distribution with a python script that is provided on the hapFLK webpage^1^. A *q*-value threshold of 0.05 was applied to limit the number of false positives.

### Gene Annotation

Gene annotation of the identified regions under possible selection was completed using NCBI’s Genome Data Viewer^2^ on the chicken genome version Gallus gallus 5.

## Results

### Population Stratification

**FIGURE 1:**
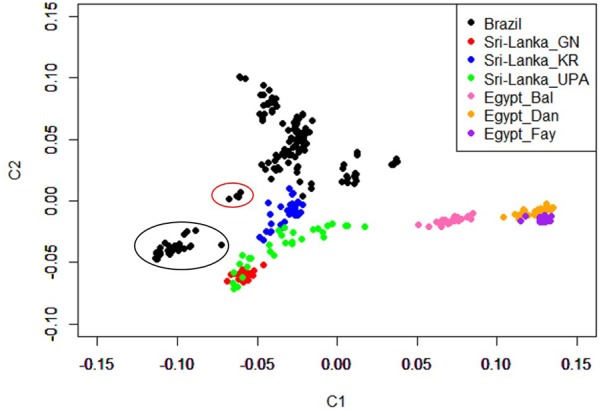
Multi-dimensional scaling (MDS) plot showing distinct sampling populations from three countries; Brazil, Sri Lanka, and Egypt. MDS plot was constructed using genomic distances to examine population stratification. The Cochinchina and Brahma Brazil breeds (black circled) and Sedosa (red circled) clustered separate and away from the rest of the eight Brazilian breeds/ecotypes.

**FIGURE 2:**
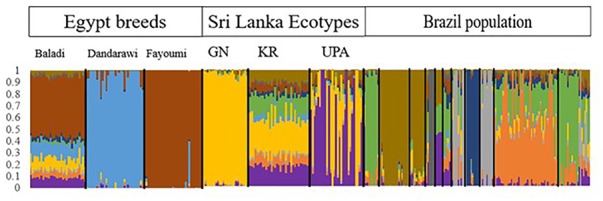
The admixture plot showing mixed ancestry among individuals and populations. The Brazil breeds/ecotypes from left to right; Shamo, Brahma, Cochinchina, Bakiva, Sedosa, Bantham, Suri, Brazilian musician, Ketros oceania, Backyard Giant Indian, and Backyard.

**FIGURE 3:**
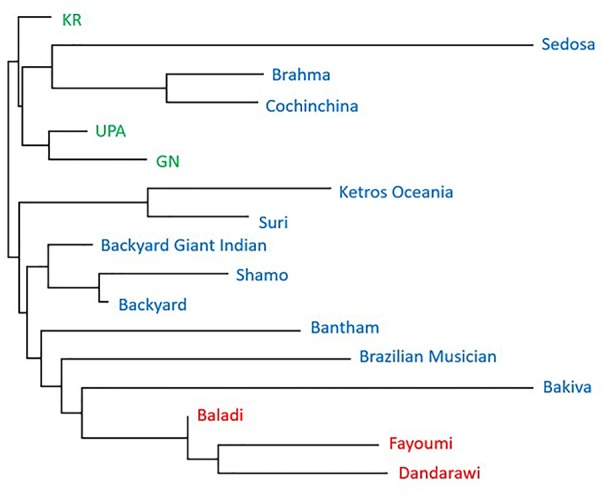
Reynolds’ genetic distances population tree of three Sril Lanka ecotypes (Green), three Egyptian breeds (Red), and eleven Brazilian breeds/ecotypes (Blue).

The MDS plot in Figure 1 shows distinct separation among ecotypes from the three countries and separation of Brazilian and Sri Lankan ecotypes from the Egyptian ecotypes. The Brazilian breeds, Cochinchina and Brahma (black circled) and Sedosa (red circled) are separated from the rest of the Brazilian breeds/ecotypes, but closer to Sri Lanka ecotypes. The admixtures analysis based on the SNP genotyping calls showed evidence of shared ancestry among breeds/ecotypes within each country and limited across countries (Figure 2). Although the Brazilian breeds/ecotypes were sampled from one location, admixture results revealed limited crossover among breed/ecotypes. The phylogenetic tree based on Reynolds’ distances with all the SNPs that passed quality control is shown in Figure 3. Here, the Sri Lankan ecotypes were separated from Egyptian breeds and some Brazilian breeds/ecotypes grouped in sub-trees. This is consistent with MDS plot. The Brazilian breeds, Cochinchina and Brahma, that are historically known to originate from Asia are grouped in one sub-tree with the Sri Lankan ecotypes.

### Fst Analyses

**FIGURE 4:**
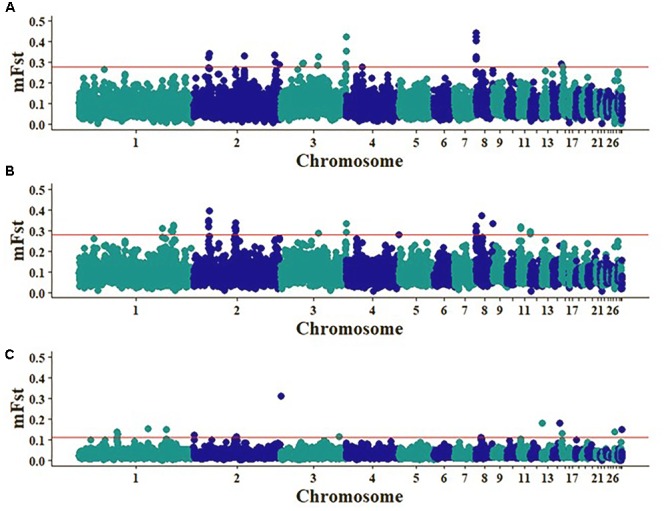
Pairwise Fst analyses to detected regions under possible selection: Sri Lanka vs. Egypt **(A)**, Brazil vs. Egypt **(B)**, Sri Lanka vs. Brazil **(C)**. Red line indicates the upper 0.2% of the empirical distribution of the window mFst values.

The Fst analyses for the comparisons between Brazil or Sri Lanka vs. Egypt generally indicated the strongest peaks on chromosomes 2, 3, and 8 (mFst > 0.28) (Figure 4). A total of two regions were detected only in the Brazil vs. Egypt comparison, on chromosomes 2 (71.85–71.95 Mb) and 8 (10.45–10.55 Mb) that contained the MicroRNA 6545 and *TRMT1L* (tRNA methyltransferase 1 like) genes, respectively. For the Sri Lanka vs. Egypt comparison, a region on chromosome 3 (64.65–64.75 Mb) was detected and contained the *HS3ST5* gene. There were also common regions between the two analyses of chickens from Brazil or Sri Lanka vs. Egypt. A total of three common regions were identified on chromosome 2 (25.25–25.35 Mb; 25.35–25.45 Mb; and 26.15–26.25 Mb) with 38, 40, and 45 SNPs, respectively. Chromosomes 3 and 8 had each one common region of 111.25–111.35 Mb and 650–750 Kb with 4 and 44 SNPs, respectively. The Brazil vs. Sri Lanka comparison had generally the lower mFst values.

### Genes Under Putative Selection Within Egyptian, Sri Lankan, and Brazilian Populations

**FIGURE 5:**
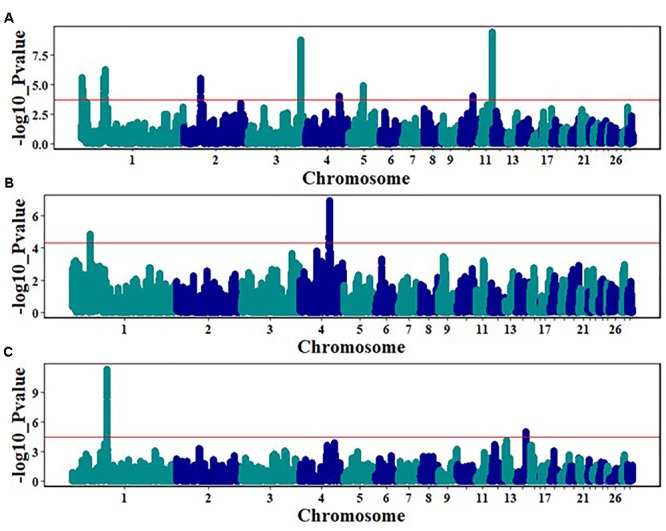
HapFLK analysis over the entire genome across breeds/ecotypes in three country populations: red line indicates the upper 0.05% of hapFLK distribution, for **(A)** within Egyptian breeds/ecotype, **(B)** within Sri Lanka ecotypes, and **(C)** with Cochinchina and Brahma Brazil breeds.

The hapFLK statistic is an extension of FLK, accounts for the haplotype information and hierarchical structure (Fariello et al., 2013; Servin et al., 2013) and greatly improves the power of detection of selection signatures that may be occurring across several populations. HapFLK analyses revealed significant unique selection signals within Sri Lankan, Egyptian, and Brazilian chicken populations. Eight significant regions on chromosomes 1 (1.71–2.72 Mb; 43.05–46.79 Mb), 2 (38.74–38.96 Mb), 3 (102.39–103.09 Mb), 4 (71.24–71.34 Mb), 5 (28.61–29.14 Mb), 10 (14.06–14.09 Mb), and 11 (18.79–20.20 Mb) were detected as strong selection signatures across the Egyptian breeds (Figure 5A). Multiple genes, with a majority of them such as Suppressor of cytokine signaling 2 (*SOCS2*), Eomesodermin (*EOMES*) and Nuclear factor of activated T-cells 5 (*NFAT5*) are involved in the immune system were identified within the regions under selection (Tables 1, 2), but to date there were no annotated genes within the regions on chromosomes 4 and 10. Two regions with strong selection signals were detected on chromosomes 1 (34.44–34.53 Mb) and 4 (61.18–62.15 Mb) across the Sri Lankan chicken ecotypes (Figure 5B). One gene was identified within the chromosome 1 region, while 18 genes, including genes involved in the immune system such as Toll like receptor 3 (*TLR3*) and Nuclear factor kappa B subunit 1 (*NFKB1*) were identified within the chromosome 4 selection region (Tables 3, 4). In addition to immune response genes, hapFLK analyses revealed genes associated with production traits in the regions under selection across Egypt and Sri Lanka chicken populations. Genes such as SNRPF, MRPL42, and ACSF3 on chromosomes 1 and 11 (Table 2) were identified across the Egypt populations, whilst MTNR1A and CYP4V2 on chromosome 4 (Table 4) were identified across the Sri Lanka populations.

**Table 1:** Putative selective signatures identified across Egyptian breeds in the hapFLK analysis.

		Number	Peak	Peak	Number
Chr	Position (Mb)	Sig SNP	*P*-value	*Q*-value	of genes
1	1.71–2.72	260	2.38 × 10^-6^	1.47 × 10^-3^	21
1	43.05–46.79	493	5.26 × 10^-7^	5.68 × 10^-4^	45
2	38.74–38.96	97	3.00 × 10^-6^	1.73 × 10^-3^	2
3	102.39–103.09	370	1.59 × 10^-9^	2.20 × 10^-5^	3
4	71.24–71.34	15	9.35 × 10^-5^	2.59 × 10^-2^	–
5	28.61–29.14	133	1.19 × 10^-5^	5.02 × 10^-3^	15
10	14.06–14.09	35	9.18 × 10^-5^	2.55 × 10^-2^	–
11	18.79–20.20	709	3.25 × 10^-10^	2.20 × 10^-5^	66

**Table 2:** List of genes in the identified putative selection signatures among Egyptian breeds.

Chr	Position window (Mb)	Gene	Gene name	Chr	Position window (Mb)	Gene	Gene name
1	1421469–1841513	EXOC4	Exocyst complex component 4	1	43288463–43293178	DUSP6	Dual specificity phosphatase 6
1	2290782–2647135	PLXNA4	Plexin A4	1	45665323–45666646	SNRPF	Small nuclear ribonucleoprotein polypeptide F
1	2002502–2187494	CHCHD3	Coiled-coil-helix-coiled-coil-helix domain containing 3	1	46695788–46706502	SLC25A3	Solute carrier family 25 member 3
1	2713344–2713453	MIR6621	microRNA 6621	1	46669629–46691852	TMPO	Thymopoietin
1	1848706–1850489	LOC101752016	G2/M phase-specific E3 ubiquitin-protein ligase-like	1	46033709–46057314	NEDD1	Neural precursor cell expressed, developmentally down-regulated 1
1	2048592–2050795	LOC107054335	PHD finger protein 7-like	1	45703687–45718329	LTA4H	Leukotriene A4 hydrolase
1	2058419–2061475	LOC107054228	Pre-mRNA-splicing factor CWC22 homolog	1	45535393–45550179	METAP2	Methionyl aminopeptidase 2
1	1916410–1918088	LOC107053764	Serine/arginine repetitive matrix protein 2-like	1	44903727–44979578	CRADD	CASP2 and RIPK1 domain containing adaptor with death domain
1	43966142–43979647	KERA	Keratocan	1	43326701–43370334	POC1B	POC1 centriolar protein B
1	44020264–44058900	DCN	Decorin	1	45241553–45243426	SNAPC3	Small nuclear RNA activating complex polypeptide 3
1	43987396–43999595	LUM	Lumican	1	46756364–47168914	ANKS1B	Ankyrin repeat and sterile alpha motif domain containing 1B
1	43925158–43947733	EPYC	Epiphycan	1	46718009–46745676	APAF1	Apoptotic peptidase activating factor 1
1	45323970–45368412	NR2C1	Nuclear receptor subfamily 2, group C, member 1	1	44597766–44618159	PLEKHG7	Pleckstrin homology and RhoGEF domain containing G7
1	44841388–44860420	UBE2N	Ubiquitin conjugating enzyme E2 N	1	44892703–44894378	SOCS2	Suppressor of cytokine signaling 2
1	45669067–45679558	AMDHD1	Amidohydrolase domain containing 1	1	46334585–46334684	MIR135A2	MicroRNA 135a-2
1	45597984–45634269	NTN4	Netrin 4	1	46258844–46258934	MIR1691	MicroRNA 1691
1	45552791–45570147	USP44	Ubiquitin specific peptidase 44	1	44585149–44588135	C12orf74	Chromosome 12 open reading frame 74
1	45439097–45489126	VEZT	Vezatin, adherens junctions transmembrane protein	1	46516656–46518848	LOC101747799	E3 ubiquitin-protein ligase ICP0-like
1	45371070–45438999	FGD6	FYVE, RhoGEF and PH domain containing 6	1	45694932–45698789	LOC107052957	Leukotriene A-4 hydrolase-like
1	45308442–45315920	NDUFA12	NADH:ubiquinone oxidoreductase subunit A12	1	44901482–44905021	LOC107052933	Translation initiation factor IF-2-like
1	45161626–45287027	TMCC3	Transmembrane and coiled-coil domain family 3	1	43397796–43455131	ATP2B1	ATPase plasma membrane Ca^2+^ transporting 1
1	45133308–45152944	CEP83	Centrosomal protein 83	1	44618268–44689672	EEA1	Early endosome antigen 1
1	45063882–45133354	PLXNC1	Plexin C1	1	44816343–44840242	NUDT4	Nudix hydrolase 4
1	44398412–44400942	BTG1	BTG anti-proliferation factor 1	1	46708852–46717870	IKBIP	IKBKB interacting protein
1	44870317–44877531	MRPL42	Mitochondrial ribosomal protein L42	5	28954792–28959688	LOC423277	Galectin-related protein-like
1	45679230–45694836	HAL	Histidine ammonia-lyase	5	29050393–29057204	FAM71D	Family with sequence similarity 71, member D
1	45796319–45887288	CDK17	Cyclin dependent kinase 17	5	28817094–28868644	PLEKHH1	Pleckstrin homology, MyTH4 and FERM domain containing H1
1	45759159–45793337	ELK3	ELK3, ETS transcription factor	5	28894687–28902644	VTI1B	Vesicle transport through interaction with t-SNAREs 1B
1	45920347–46028422	CFAP54	Cilia and flagella associated protein 54	5	29066540–29333342	GPHN	Gephyrin
2	38923901–38967870	CMC1	C-X 9-C motif containing 1	5	28869790–28876697	PIGH	Phosphatidylinositol glycan anchor biosynthesis class H
2	38739416–38744143	EOMES	Eomesodermin	5	102733739–102742985	TDRD15	Tudor domain containing 15
3	102659050–102693303	APOB	Apolipoprotein B	5	28877624–28894686	ARG2	Arginase 2
3	102386988–102495414	LDAH	Lipid droplet associated hydrolase	5	28983479–28991363	ATP6V1D	ATPase H+ transporting V1 subunit D
5	28973589–28983409	EIF2S1	Eukaryotic translation initiation factor 2 subunit alpha		28960146–28968627	PLEK2	Pleckstrin 2
5	28992145–29040405	MPP5	Membrane palmitoylated protein 5	5	28761838–28794172	TMEM229B	Transmembrane protein 229B
5	28910734–28954526	ZFYVE26	Zinc finger FYVE-type containing 26	5	28405397–28748814	RAD51B	RAD51 paralog B
5	28902870–28910030	RDH12	Retinol dehydrogenase 12 (all-trans/9-cis/11-cis)	11	20154497–20159550	TMEM231	Transmembrane protein 231
11	19157119–19158772	CHTF8	Chromosome transmission fidelity factor 8	11	20160472–20168822	CHST6	Carbohydrate sulfotransferase 6
11	19174900–19176268	NIP7	NIP7, nucleolar pre-rRNA processing protein	11	20146831–20154681	GABARAPL2	GABA type A receptor associated protein like 2
11	19004528–19008784	CDK10	Cyclin dependent kinase 10	11	19173208–19174878	COG8	Component of oligomeric golgi complex 8
11	20121004–20145653	ADAT1	Adenosine deaminase, tRNA specific 1	11	19163629-19168951	SNTB2	Syntrophin beta 2
11	20108842–20121014	KARS	Lysyl-tRNA synthetase	11	19158792–19163525	UTP4	UTP4, small subunit processome component
11	19994665–20039204	AP1G1	Adaptor related protein complex 1 gamma 1 subunit	11	19089879–19093651	DEF8	Differentially expressed in FDCP 8 homolog
11	19961317–19964798	DHODH	Dihydroorotate dehydrogenase (quinone)	11	20088785–20104757	CHST4	Carbohydrate (*N*-acetylglucosamine 6-*O*) sulfotransferase 4
11	19949492–19960006	DHX38	DEAH-box helicase 38	11	19986527–19994083	ATXN1L	Ataxin 1 like
11	19316519–19321831	PSMD7	Proteasome 26S subunit, non-ATPase 7	11	18805694–18846630	ACSF3	Acyl-CoA synthetase family member 3
11	19283664–19316100	WWP2	WW domain containing E3 ubiquitin protein ligase 2	11	19169788–19172777	VPS4A	Vacuolar protein sorting 4 homolog A
11	19192249–19206013	CYB5B	Cytochrome b5 type B	11	19064480–19082896	TCF25	Transcription factor 25
11	18946166–18976689	SPG7	SPG7, paraplegin matrix AAA peptidase subunit	11	20039684–20070358	PHLPP2	PH domain and leucine rich repeat protein phosphatase 2
11	19123969–19151197	TMCO7	Transmembrane and coiled-coil domains 7	11	19972783–19984346	ZNF821	Zinc finger protein 821
11	19281166–19283712	NOB1	NIN1/PSMD8 binding protein 1 homolog	11	18926659–18926760	MIR1785	MicroRNA 1785
11	19105097–19107786	URAH	5-hydroxyisourate hydrolase	11	18874320–18874423	MIR1560	MicroRNA 1560
11	19093976–19096929	DBNDD1	Dysbindin (dystrobrevin binding protein 1) domain containing 1	11	19730962–19731058	MIR1699	MicroRNA 1699
11	19057744–19063946	SPIRE2	Spire-type actin nucleation factor 2	11	19009229–19010941	SPATA2L	Spermatogenesis associated 2 like
11	19022586–19055008	FANCA	Fanconi anemia complementation group A	11	18848471–18853003	CDH15	Cadherin 15
11	19015485–19022670	ZNF276	Zinc finger protein 276	11	19944361–19949539	PMFBP1	Polyamine modulated factor 1 binding protein 1
11	18990705–18993412	SULT2B1L1	Sulfotransferase family cytosolic 2B member 1-like 1	11	20069985–20076137	MRVLDC3	MARVEL domain-containing protein 3
11	18983791–18989285	CPNE7	Copine VII	11	19086099–19087230	LOC107054334	Translation initiation factor IF-2-like
11	18857696–18939049	ANKRD11	Ankyrin repeat domain 11	11	19255421–19262314	LOC101750188	Envelope glycoprotein gp95-like
11	18993512–18997373	DPEP1	Dipeptidase 1 (renal)	11	19940111–19944311	LOC415872	Inner nuclear membrane protein Man1-like
11	18999512–19003217	CHMP1A	Charged multivesicular body protein 1A	11	18853081–18856750	SLC22A31	Solute carrier family 22 member 31
11	19010926–19015340	VPS9D1	VPS9 domain containing 1	11	19965154–19972318	IST1	IST1, ESCRT-III associated factor
11	19007943–19008052	MIR6667	MicroRNA 6667	11			
11	19114383–19123146	CDH1	Cadherin 1	11	19084582–19085526	MC1R	Melanocortin 1 receptor
11	19107862–19113818	CDH3	Cadherin 3	11	20106176–20109169	TERF2IP	TERF2 interacting protein
11	19310301–19310395	MIR140	MicroRNA 140		19176609–19179130	TMED6	Transmembrane p24 trafficking protein 6
11	19321916–19834198	ZFHX3	Zinc finger homeobox 3	11	19152159–19155850	HAS3	Hyaluronan synthase 3
11	19278734–19280589	NQO1	NAD(P)H quinone dehydrogenase 1	11	19180205–19188036	TERF2	Telomeric repeat binding factor 2
11	20079229–20088689	TAT	Tyrosine aminotransferase	11	19212134–19277188	NFAT5	Nuclear factor of activated T-cells 5
11	18978071–18982325	RPL13	Ribosomal protein L13	11	19097379–19105093	GAS8	Growth arrest specific 8

**Table 3:** Putative selection signatures identified across Sri Lanka ecotypes in the hapFLK analysis.

		Number			
		significant	Peak	Peak	Number
Chrom	Position (Mb)	SNP	*P*-value	*Q*-value	of genes
1	34.44–34.53	39	1.40 × 10^-5^	2.21 × 10^-2^	1
4	61.18–62.15	469	1.21 × 10^-7^	2.10 × 10^-3^	18

**Table 4:** List of genes in the identified putative selection signatures among Sri Lanka ecotypes.

Chr	Position window	Gene	Gene name
1	34429461–34736907	GRIP1	Glutamate receptor interacting protein 1
4	61704266–61714229	TLR3	Toll like receptor 3
4	61175278–61232965	NFKB1	Nuclear factor kappa B subunit 1
4	62021985–62071062	MTNR1A	Melatonin receptor 1A
4	62099614–62202082	FAT1	FAT atypical cadherin 1
4	61451128–61473588	PDLIM3	PDZ and LIM domain 3
4	61300211–61333975	UBE2D3	Ubiquitin conjugating enzyme E2 D3
4	61433970–61441668	CCDC110	Coiled-coil domain containing 110
4	61337821–61362524	SLC9B2	Solute carrier family 9 member B2
4	61774102–61783297	F11	Coagulation factor XI
4	61714653–61743467	FAM149A	Family with sequence similarity 149 member A
4	61486294–61629945	SORBS2	Sorbin and SH3 domain containing 2
4	61426124–61433976	C4H4ORF47	Chromosome 4 open reading frame, human C4orf47
4	61379229–61415856	CENPE	Centromere protein E
4	61362628–61376776	BDH2	3-hydroxybutyrate dehydrogenase, type 2
4	61334093–61340503	CISD2	CDGSH iron sulfur domain 2
4	61229303–61288017	MANBA	Mannosidase beta
4	61746761–61759707	CYP4V2	Cytochrome P450 family 4 subfamily V member 2

There were no strong selection signals across the eleven Brazilian breeds/ecotypes, but two regions with strong signals were detected across the two Brazilian breeds with Asian ancestry, Cochinchina and Brahma on chromosomes 1 and 14 (Figure 5C). Three genes were identified within the selection signature region on chromosome 1 and there were no annotated genes within the chromosome 14 region (Tables 5, 6). No selection signals were detected across the rest of the nine Brazilian breeds/ecotypes (results not shown). None of the selection signature regions from the hapFLK in any country (Egypt, Sri Lanka, and Brazil) populations were consistent with Fst analyses.

**Table 5:** Putative selection signatures identified across Cochinchina and Brahma Brazilian breeds in the hapFLK analysis.

		Number
		significant	Peak	Peak	Number
Chrom	Position (Mb)	SNP	*P*-value	*Q*-value	of genes
1	65.52–66.12	299	5.25 × 10^-12^	4.42 × 10^-7^	3
14	10.47–10.53	45	8.87 × 10^-6^	1.69 × 10^-2^	–

**Table 6:** List of genes in the identified putative selection signatures among Cochinchina and Brahma Brazilian chicken breeds.

Chr	Position window	Gene	Gene name
1	65660324–66227364	SOX5	SRY-box 5
1	65898377–65898486	MIR6608-2	microRNA 6608-2
1	65891957–65892066	MIR6608-1	microRNA 6608-1

## Discussion

The admixture of populations in the three countries indicates mixed genetic backgrounds of the chickens (Figure 3). The overlap across ecotypes/breeds within individual countries could be due to unrestricted inter-mating among chickens of different genetic backgrounds, resulting in chickens with ancestors from different groups that eventually contribute to the shared ancestry. The other factor that might contribute to the admixture within and across the respective countries could be due to movement of birds through trading. Although chickens were sampled from one location, Porto Ferreira in Brazil, it is surprising that there was more admixture and more discrete breeds in the Brazil population, unlike Egypt and Sri Lanka populations. Moreover, the Brazilian breeds/ecotypes clustered closer to the Sri Lankan ecotypes (Figures 1, 3). This is, however, not surprising because chickens in Brazil are not indigenous and are reported to have been imported from Asia (Komiyama et al., 2004). The Reynolds’ genetic distances population tree compliments the stratification by the MDS plot and admixture of the populations. The Egyptian breeds are within their own sub-tree and appear to have some shared ancestry with some Asian breeds as revealed by the admixture plot. The indication of shared ancestry is in agreement with previous findings which reported that Egyptian local/native breeds/ecotypes originated from Asia or the Indian sub-continent (Elferink et al., 2012; Elkhaiat et al., 2014; Eltanany and Hemeda, 2016).

The MDS results allowed the analyses to be performed on a case/control basis, with environmental/climatic conditions of the three countries as the proxy phenotype to allow the results to be viewed as regions of the genome under possible selection for environmental tolerance/adaptation by the local chicken populations of each of the three countries. The Fst results indicated possible selection signatures on chromosomes 2 and 8 for the Brazil vs. Egypt comparison, and on chromosome 3 for the Sri Lanka vs. Egypt comparison and common differences between Arid (Egypt) and Tropical (Sri Lanka and Brazil). The two genes, TRMT1L and MicroRNA 6545 detected in regions for the Brazil vs. Egypt comparison could suggest chicken adaptation and survival in hot conditions. *TRMT1L* catalyzed tRNA modification is required for redox homeostasis to ensure proper cellular proliferation and oxidative stress survival. Cells that are deficient in the *TRMT1L* will exhibit a decrease in proliferation rates, alteration in protein synthesis and perturbation in redox homeostasis including hypersensitivity to oxidizing agents (Dewe et al., 2017). The second gene, *MicroRNA 6545*, is reported to be involved in reproductive processes and embryogenesis, including TGF-β and Wnt that specifies the neutral fate of the blastodermal cells (Shao et al., 2012). For the Sri Lanka vs. Egypt comparison, a gene, *HS3ST5* that could be important in immune response was detected. *HS3ST5* is involved in immunity and defense molecular functions (Szauter et al., 2011). Although we did not detect annotated genes in the common regions between the two analyses of chickens from Brazil or Sri Lanka vs. Egypt, these regions could present recent important selection signatures that could enable chicken survival in either the tropics or arid conditions. The common genomic regions of chickens from Sri Lanka or Brazil when compared to Egypt could indicate exposure of chickens from Sri Lanka and those from Porto Ferreira (Brazil) to same environmental conditions and they may have evolved similar selection signatures for adaptation and survival.

The identification of genomic regions that may be under both artificial and natural selection could help identify possible selection signatures across breeds/ecotypes within a country. Several genomic regions with putative selection were identified in the current study using the hapFLK method across Egyptian and Sri Lankan breeds and ecotypes, respectively. The hapFLK analyses identified several regions under selection on chromosomes 1, 2, 3, 4, 5, 10, and 11, across the three Egyptian breeds; Fayoumi, Dandarawi, and Baladi (Figure 5A and Table 1). Some genes detected in the genomic regions under selection across the Egyptian chickens are reported to be involved in the modulation of growth (Bolamperti et al., 2013), and the immune system (Szczesny et al., 2014; Zhang et al., 2018) and others could possibly be important in thermal/heat tolerance. These genes could be relevant in the adaptation of the Egyptian chickens to the arid hot dry conditions. One notable gene in a region under selection, on chromosome 2 is the *SOCS2*. Suppressor of cytokine signaling (SOCS) proteins generally play vital roles in the feedback inhibition of cytokine receptor signaling (Larsen and Röpke, 2002). The *SOCS2* gene is a multifunctional protein that is involved in growth hormone signaling through cytokine-dependent pathways and the JAK/STAT pathway (Metcalf et al., 2000; Rico-Bautista et al., 2006). This gene is important in the regulation of several biological processes that control growth, development, immune function, homeostasis (Rico-Bautista et al., 2006), and has been hypothesized to have an effect on breast meat yield during heat stress (Van Goor et al., 2015). The region on chromosome 2 under selection contains two genes, and one of the genes, *EOMES* is also important in the immune system. The *EOMES* is one of the two T-box proteins expressed in the immune system and are responsible with driving the differentiation and function of cytotoxic innate lymphocytes such as the natural killer (NK cells). NK cells are endowed with cytotoxic properties and contribute to the early defense against pathogens and immunosurveillance of tumors (Zhang et al., 2018). The regions under selection on chromosome 11 contains 66 annotated genes, with some genes involved in immune response. One of the genes, *NFAT5* is required for TLR-induced responses to pathogens, and previous studies have shown that TLR-induced *NFAT5*-regulated genes such as TNF-α play a vital role in inflammatory responses (Buxadé et al., 2012; Tellechea et al., 2017). We have reported only a few genes plus their associated roles/functions in regard to the regions under selection across the Egyptian breeds. Most of the genes in these regions on the different chromosomes (1, 2, 3, 5, and 11) could play vital roles in the adaptation mechanisms to enable the survival of the Egyptian chicken breeds in the hot arid climatic conditions. Although we did not detect any annotated genes in the regions under selection on chromosomes 4 and 10, it is important to note that these could be recent possible selection signatures for the Egyptian breeds to their climate. In other parallel studies, it has been shown that domesticated animals often develop physiological and genetic adaptations when encountered with harsh or new environments such as hypoxia (Ramirez et al., 2007; Storz et al., 2010). A study conducted on Tibetan chickens that primarily live at high altitudes of between 2,200 and 4,100 m revealed several candidate genes that are involved in the calcium signaling pathway to possibly enable them adapt to hypoxia (Wang et al., 2015). There were two regions under selection on chromosomes 1 and 4 across the Sri Lanka ecotypes. Like the selection in the Egyptian breeds, the region under selection on chromosome 4 of the Sri Lanka ecotypes contain several genes and two of them, Toll like receptor 3 (*TLR3*) and Nuclear factor kappa B subunit 1 (*NFKB1*) are important in the immune system. A TLR signaling pathway is an innate immune defense mechanism against pathogen attack in both vertebrates and invertebrates. *TLR3* in chickens is orthologous to its mammalian counterpart (Kannaki et al., 2010), and together with *TLR7* it is known in the recognition of RNA virus encoded pathogen associated molecular patterns (PAMPs) (Akira, 2001). *TLR3* are able to recognize and bind to double-stranded RNA intermediates that are produced during viral replication (Iqbal et al., 2005), and the end product of its signaling pathway is the production of anti-viral type I inferno (IFN)-α and -β (Guillot et al., 2005). Another important gene, *NFKB1* could also be of importance to the survival of Sri Lanka chicken ecotypes in the tropical hot humid climate climatic conditions of Sri Lanka. *NFKB* transcription factors are important in immunity and inflammation (Hayden and Ghosh, 2008). TLR are activated by binding to the PAMPs that in turn initiates MAPK- or nuclear factor kappa B (*NFkB*) dependent cascades that leads to a proinflammatory response, resulting in the secretion of antibacterial substances, such as β-defensins and cytokines (Kogut et al., 2006). *NFKB* proteins are also involved in a wide range of processes, including; cell development, growth and survival, proliferation and are also involved in many pathological conditions (Morgan and Liu, 2011). Sri Lanka has hot humid climatic conditions that besides being favorable for pathological infection to livestock, also presents challenging conditions like heat stress, especially during a drought that requires the animal to adapt to such conditions. Challenges like heat stress result in the production of ROS that are produced by a variety of cellular processes. *NFKB*-regulated genes are vital in regulating the amount of ROS in cells (Morgan and Liu, 2011). The ROS have several stimulatory and inhibitory roles in *NFKB* signaling.

Chicken survival in challenging environments involves different adaptation mechanisms, among which is the ability to perform under harsh conditions. The current study indicated selection signatures with genes associated with production traits in both Egypt and Sri Lanka populations. For Egypt populations, we identified *MRPL42* which is a candidate gene associated with breast yield under heat stressed chickens. The *MRPL42* gene is vital in DNA synthesis, transcription, RNA processing and translation (Van Goor et al., 2015). Another gene *ACSF3*, belonging to the ACSF gene family is reported to be correlated to egg laying performance in chickens (Tian et al., 2018). For Sri Lanka chicken populations, the *CYP4V2* gene associated with control of fat deposition in chickens was identified on chromosome 4 of the region under selection (Claire D’Andre et al., 2013). Because local chickens are mostly free range and exposed to high humid hot conditions in developing countries, such as Sri Lanka, it could be vital for chickens to control the depositions of fat as an adaption mechanism.

There were no regions of selection across all the eleven Brazilian breeds/ecotypes, but we detected possible regions of selection across two breeds, Cochinchina and Brahma, known to have Asian ancestry, on chromosomes 1 and 4. However, these regions didn’t overlap with regions under selection across the Asian Sri Lankan ecotypes. This could be due to the fact that chickens were introduced to Brazil from Asia over a few hundred years ago, and possibly because of the differences in climatic conditions between Porto Ferreira, Sao Paolo and Sri Lanka. The chicken genomes from these locations could have been modified to enable chicken adaptation and survival in the respective changing climates.

There is clear evidence that chickens, particularly the domestic fowl, were kept in Egypt for thousands of years and this is dated back to 1840 B.C (Coltherd, 1966). For other traditional breeds such as Fayoumi and Dandarawi, studies based on mitochondrial (mtDNA) sequence variation have shown that these Egyptian indigenous breeds could have roots in Indian subcontinent and southwest Asia (Elkhaiat et al., 2014; Eltanany and Hemeda, 2016), because Egypt was an entry route of Indian chickens to Africa. In spite of the fact that Egyptian chicken breeds might have Asian origin, none of the regions under selection was shared between Egyptian breeds and Sri Lanka ecotypes. Asian chicken breeds could have been imported to Egypt over thousands of years ago, and because of the difference in climatic conditions; hot arid and hot humid for Egypt and Sri Lanka, respectively, chickens in the two climatic conditions developed different adaptation mechanisms to survive in the different climates.

The two methods, Fst and hapFLK, did not detect any overlapping regions, and we noted that hapFLK detected more selection signals with several important genes compared to Fst. HapFLK approach has been reported by previous simulation studies to have the ability to greatly increase the detection power of selection signatures occurring across several populations (Bonhomme et al., 2010; Fariello et al., 2013). Due to this, were able to detect several regions under selection; within Egypt and Sri Lanka populations with hapFLK that were not detected by the Fst analyses. HapFLK considers the hierarchical structure of the population and this improves the detection power of soft sweeps.

## Conclusion

There is evidence of stratification and admixture, particularly among breeds/ecotypes within each country’s populations. The Fst differences between Sri Lanka and Egypt populations could indicate the differences in the chicken adaptations due to the different climatic conditions in the two countries. The low Fst values between Sri Lanka and Brazil could possibly be due to common shared ancestry of Asian origin over a few years ago rather than climate. This might change with the continuous changes in climatic conditions where local Brazilian chickens from Porto Ferreira, Sao Paolo region might develop certain genome modification to adapt to the climate. For hapFLK analyses, there were no common regions under selection among breeds/ecotypes across the populations from the three countries. This could indicate climatic specific selection signals that have enabled those chickens to develop adaptation mechanisms in response to their respective climatic conditions. In that regard, Sri Lanka and Egypt chicken ecotypes/breeds have developed mechanisms to survive in their humid and dry hot climates.

## Data Availability Statement

The link to the data access: https://www.animalgenome.org/repository/pub/ISU2018.0416/. It is in the NRSP-8, Bioinformatics data repository.

## Author Contributions

All authors listed have made a substantial, direct and intellectual contribution to the work, and approved it for publication.

## Conflict of Interest Statement

The authors declare that the research was conducted in the absence of any commercial or financial relationships that could be construed as a potential conflict of interest.

## Footnotes

1 https://forge-dga.jouy.inra.fr/documents/588

2 https://www.ncbi.nlm.nih.gov/genome/gdv/
